# Effects of Wenxin Keli on the Action Potential and L-Type Calcium Current in Rats with Transverse Aortic Constriction-Induced Heart Failure

**DOI:** 10.1155/2013/572078

**Published:** 2013-11-11

**Authors:** Yu Chen, Yang Li, Lili Guo, Wen Chen, Mingjing Zhao, Yonghong Gao, Aiming Wu, Lixia Lou, Jie Wang, Xiaoqiu Liu, Yanwei Xing

**Affiliations:** ^1^Shenyang Pharmaceutical University, Shenyang, Liaoning 110016, China; ^2^Guang'anmen Hospital, China Academy of Chinese Medical Sciences, Beijing 100053, China; ^3^Institute of Geriatric Cardiology of Chinese PLA General Hospital, Beijing 100853, China; ^4^The Key Laboratory of Chinese Internal Medicine of the Ministry of Education, Dongzhimen Hospital Affiliated to Beijing, University of Chinese Medicine, Beijing 100700, China

## Abstract

*Objective*. We investigated the effects of WXKL on the action potential (AP) and the L-type calcium current (*I*_Ca-L_) in normal and hypertrophied myocytes. *Methods*. Forty male rats were randomly divided into two groups: the control group and the transverse aortic constriction- (TAC-) induced heart failure group. Cardiac hypertrophy was induced by TAC surgery, whereas the control group underwent a sham operation. Eight weeks after surgery, single cardiac ventricular myocytes were isolated from the hearts of the rats. The APs and *I*_Ca-L_ were recorded using the whole-cell patch clamp technique. *Results*. The action potential duration (APD) of the TAC group was prolonged compared with the control group and was markedly shortened by WXKL treatment in a dose-dependent manner. The current densities of the *I*_Ca-L_ in the TAC group treated with 5 g/L WXKL were significantly decreased compared with the TAC group. We also determined the effect of WXKL on the gating mechanism of the *I*_Ca-L_ in the TAC group. We found that WXKL decreased the *I*_Ca-L_ by accelerating the inactivation of the channels and delaying the recovery time from inactivation. *Conclusions*. The results suggest that WXKL affects the AP and blocked the *I*_Ca-L_, which ultimately resulted in the treatment of arrhythmias.

## 1. Introduction

Cardiovascular diseases (CVD) are the most common threat to human health worldwide. From 1999 to 2009, the relative rates of death attributable to CVD decreased by 32.7%. However, CVD still accounted for 32.3% of deaths in the United States in 2009. Thus, the burden of this disease remains high. Epidemiological data show that an estimated 5.1 million Americans greater than 20 years of age exhibited heart failure from 2007 to 2010. Projections show that the prevalence of heart failure by 2030 will increase by 25% from the 2013 estimates [1]. In many cardiovascular diseases, cardiac hypertrophy is a common pathological process, and the disorder of the heart rhythm that is induced by cardiac hypertrophy is the most common cause of sudden cardiovascular death. A previous study indicates that 36% of the 690 athletes died as a result of cardiac hypertrophy [2]. This high incidence of cardiac hypertrophy has caused widespread concern, and these data highlight the importance of finding suitable drugs for treating cardiac hypertrophy-induced arrhythmias.

The currently electrophysiological research on cardiac hypertrophy mainly focuses on changes in the action potentials and the related ionic mechanisms. In recent years, the role of Ca signalling in cardiac myocytes was studied with respect to electrophysiology, such as the effect of Ca signalling on arrhythmias and action potentials [3]. Ca^2+^ enters the cell via the L-type Ca^2+^ current, and the sarcoplasmic reticulum (SR) releases Ca^2+^ via the ryanodine receptor (RyR) to increase the intracellular calcium concentration ([Ca^2+^]i) and thus activate cardiac contraction. However, the overloading of the SR with Ca^2+^ can induce arrhythmias [4]. Blocking Ca^2+^ channel and reducing the Ca^2+^ overload will thus benefit the treatment of arrhythmias and heart failure. The most important finding in animal models of left ventricular hypertrophy is the significant prolongation of the action potential duration (APD) at low but not at high frequencies. Recent model results suggest that even subtle changes in AP morphology that may result from remodeling of membrane transporter expression in disease may have major impact on the temporal waveform of Ca^2+^ transients, thus influencing tissue level electromechanical function [5]. The prolongation of the APD would increase the incidence of triggered activity and the early after-depolarization (EAD), which would result in increasing the incidence of arrhythmias [6, 7]. Using ion channel antagonist drugs to treat arrhythmias in patients with structural heart disease does not reduce their mortality [8, 9]. In addition, most antiarrhythmic drugs have shown potential lethal proarrhythmic effects that result in the aggravation of arrhythmias and potentially induce ventricular arrhythmias. Thus, there is an urgent need to develop effective and safer antiarrhythmic agents. In particular, traditional Chinese medicine has been well recognized for its antiarrhythmic potential. 

Wenxin Keli (WXKL) is a Chinese herb extract developed by Guang'anmen Hospital at the Chinese Academy of Chinese Medical Sciences and is the first antiarrhythmic Chinese medicine to be approved by the state. It is composed of five main components: *Nardostachys chinensis* Batal extract, Codonopsis, Notoginseng, amber, and *Rhizoma Polygonati*. Since its clinical application, this drug had been proven to be of benefit in the treatment of various diseases, such as cardiac arrhythmias, cardiac inflammation, and chronic heart failure [10]. Previous studies have shown that WXKL is a safe and effective treatment, which does not result in significant adverse reactions for the premature ventricular contractions caused by viral myocarditis, as evaluated by improvements in the clinical symptoms, signs, and ECG [11]. Some studies have investigated the effects of irbesartan, amiodarone, and WXKL, applied either alone or in combination, on sinus rhythm maintenance in patients with atrial fibrillation after conversion. It was shown that a combined therapy of Chinese and Western medicines exhibits a synergistic antiarrhythmia effect that improves the conversion rate of atrial fibrillation, shortens the conversion time, and avoids adverse reaction [12, 13]. A fascinating electrophysiologic study of the effects of WXKL suggests that this agent can depress the sodium channel-dependent parameters in canine isolated coronary-perfused preparations and effectively manages and prevents the induction of atrial fibrillation [14]. In addition, the action of notoginseng, which is one of the components of WXKL, was demonstrated to have antiarrhythmic properties, as demonstrated in rats with ischemia arrhythmias [15]. As a result, notoginseng further enhances the antiarrhythmic properties of WXKL. Moreover, it has been reported that WXKL exhibits beneficial effects on isoproterenol- (ISO-) induced heart failure in rats. In addition, WXKL can greatly improve ISO-induced cardiac dysfunction and protect against aconitine-induced arrhythmia in rats [16]. A recent study suggested that long-term treatment with WXKL may have seen it attenuate ischemia-induced ventricular arrhythmias in rats and inhibit *I*_Ca-L_ and *I*_*to*_ in a concentration-dependent manner, which may contribute to the observed attenuation [17].

Although a number of electrophysiological studies have analysed the use of WXKL for the treatment of cardiac hypertrophy, the effects of WXKL on the APs and *I*_Ca-L_ of normal and hypertrophied myocytes have not been reported. Thus, the goal of this study was to evaluate the effect of WXKL on the APs and *I*_Ca-L_ of normal and hypertrophic ventricular myocytes using whole-cell patch clamp recording techniques and to explore the mechanism through which WXKL benefits the treatment of cardiac hypertrophy, which would provide better insights into the effects of antiarrhythmic drugs.

## 2. Materials and Methods

### 2.1. Animals

Forty male Sprague-Dawley rats (body weight = 140–160 g), which were purchased from Vital River Experimental Animal Centre (license number SYXK (E) 200420007, Beijing, China) were randomly divided into two groups: the TAC group (*n* = 25) and the control group (*n* = 15). The TAC rats underwent transverse aortic constriction (TAC) surgery, and the control group underwent an identical procedure but without the application of the ligation. All of the following experiments conformed to the Guiding Principles for the Care and Use of Laboratory Animals issued by the National Committee of Science and Technology of China.

### 2.2. Drugs and Solutions

WXKL was provided by Shandong Buchang Pharmaceuticals Co., Ltd., China. The drug was dissolved with Ca^2+^-free Tyrode solution at concentrations of 0.5 g/L, 1 g/L, 5 g/L, 10 g/L, and 20 g/L prior to the experiment. The Ca^2+^-free Tyrode solution contained (in mmol/L) 137 NaCl, 5.4 KCl, 1.0 MgCl_2_, 0.33 NaH_2_PO_4_, 10 hydroxyethyl piperazine ethanesulfonic acid (HEPES), and 10 glucose (pH 7.35, adjusted with NaOH). The Krebs buffer (KB) solution for cell storage contained (in mmol/L) 40 KCl, 20 KH_2_PO_4_, 3.0 MgCl_2_, 70 KOH, 50 L-glutamic acid, 10 HEPES, 20 taurine, 10 glucose, and 0.5 EGTA (pH 7.35, adjusted with KOH). For the AP recordings, the internal pipette solution contained (in mmol/L) 120 K aspartate, 20 KCl, 1.0 MgCl_2_, 4.0 Na_2_ATP, 10 glucose, and 10 HEPES, and the bath solution contained (in mmol/L) 140 NaCl, 1.0 CaCl_2_, 1.0 MgCl_2_, 4 KCl, 10 HEPES, and 5 glucose. For the *I*_Ca-L_ recordings, the internal pipette solution contained (in mmol/L) 120 CsCl, 1.0 CaCl_2_, 5.0 MgCl_2_, 5.0 Na_2_ATP, 11 EGTA, 10 HEPES, and 11 glucose (pH 7.3, adjusted with CsOH), and the bath solution was the Tyrode solution supplemented with 1.8 mmol/L CaCl_2_.

### 2.3. Creation of the TAC Model

**Figure 1:**
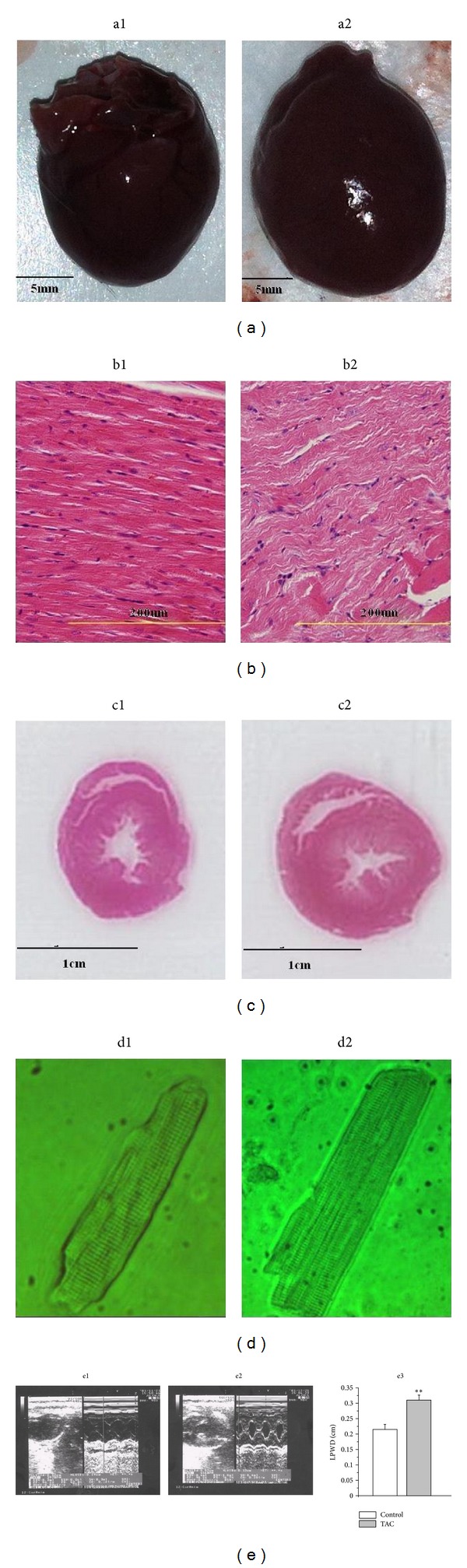
(a) Preparation of the hearts from the control group (a1) and the TAC group (a2). (b) Left ventricular apical biopsy of the control group ((b1), HE staining 400x magnification) and the TAC group ((b2), HE staining 400x magnification). (c) Pathological section of the largest cross-section of the control group (c1) and the TAC group (c2). (d) Single ventricular myocytes from the control group (d1) and the TAC group (d2). (e) Typical echocardiography images from the control group (e1) and the TAC group (e2). Eight weeks after the TAC surgery, the cardiac structure and function were measured through echocardiography. We evaluated the cardiac systolic and diastolic functions by measuring the left ventricular posterior wall thickness (LPWD) of the control group and the TAC group (c3). **P < 0.01 versus the control group.

The transverse aortic constriction (TAC) surgery was performed in male Sprague-Dawley rats as described previously [18, 19]. Briefly, the rats were anesthetised with 3% chloral hydrate (300 mg/kg, intraperitoneally). The thorax was opened, and a 4-0 silk suture was passed under the aorta between the origin of the right innominate and the left common carotid arteries. A 6 G needle was placed on the ascending aorta, and the suture was snugly tied around the needle and the aorta. The probe was then quickly removed. The skin was closed, and the rats were maintained in a heating pad until they recovered from the anesthesia. The sham-operated animals underwent an identical procedure but without the application of the ligation. After surgery, both groups were fed tap water and normal fodder in different cages for 8 weeks. To characterise the model, echocardiographic measurements were obtained 8 weeks after surgery using a Vivid 7 Dimension cardiovascular ultrasound system (GE Healthcare, Fairfield, Connecticut, United States) as described previously [20]. The detection indicator was the left ventricular posterior wall thickness (LPWD), and the parameters measured are shown in Figure 1(e).

### 2.4. Cardiac Ventricular Myocytes Isolation

Single cardiac ventricular myocytes were isolated from the hearts of the rats as previously described [21] with slight modifications. Briefly, 5 minutes after the rats were heparinised (100 U/mL 1 mL/100 g i.p.), the animals were anesthetised with 3% chloral hydrate (0.5 mL/100 g i.p). The heart was rapidly excised and mounted on the Langendorff apparatus and perfused via the aorta with oxygenated Ca^2+^-free Tyrode solution for 5 minutes and then with Ca^2+^-free Tyrode solution containing collagenase II (0.6 mg/mL, Worthington, USA), trypsin (0.24 mg/mL, Amresco, USA), and proteinase E (0.08 mg/mL, Amresco, USA) for 15–20 minutes at 37°C. Subsequently, the ventricular tissue was excised, cut into small pieces in a dish containing KB solution, and blown gently to obtain single ventricular myocytes. The cells were maintained at 4°C in KB solution until use. All of the solutions were continuously gassed with 95% O_2_ and 5% CO_2_ at 37°C. The single ventricular myocyte selected for electrophysiological measurements is rod-shaped, quiescent, Ca-tolerant, and had clear cross-striations and a smooth and glossy surface.

### 2.5. Histological Examination

The rat heart samples were cut into transverse sections and routinely stained with haematoxylin and eosin (H&E) as described previously [22]. The stained sections were examined under a light microscope (OLYMPUS BX51, Japan) and photographed at 400x magnification for morphological analysis.

### 2.6. Electrophysiological Recording

The whole-cell patch clamp technique was used to record the APs and *I*_Ca-L_ using an Axopatch 700B amplifier (Axon Instruments, USA) with the pCLAMP 9.2 software (Axon Instruments, USA). Borosilicate glass patch pipettes (resistance = 3–5 M*Ω*) were pulled using a vertical pipette puller (Narishige pp-830, Japan). The cells were maintained in external solution for 5 to 10 minutes after perfusion and the data were recorded after entering the cell for 5 minutes to stabilise the current. All of the recordings were performed at room temperature (22°C) within 25 minutes to avoid current rundown. The APs were elicited in the current-clamp mode at a rate of 1.0 Hz using 30 trains of suprathreshold current pulses. The membrane capacitance was calculated using the manual whole-cell capacitance controls on the Axopatch amplifier. The *I*_Ca-L_ was recorded in the voltage-clamp mode and elicited through step depolarisation from −40 to +50 mV in 10-mV increments for 250 ms.

### 2.7. Statistical Analysis

Off-line leak correction was performed on all of the amplitude data. The pCLAMP 9.2 software (Axon Instruments, USA) and the Origin 6.1 software (Microcal Software, USA) were used for the data acquisition and analysis. The data are presented as the mean ± SE, where *n* represents the number of cells analysed. The statistical comparisons between different groups were performed with ANOVA and Student's *t*-test. Differences with a value of *P* less than 0.05 were considered statistically significant.

## 3. Results

### 3.1. Echocardiographic and Histological Characteristics

Eight weeks after the TAC surgery, the cardiac structure and function were measured through echocardiographic and histological examinations. Compared with the control group, the heart was slightly enlarged (Figure 1(a)), and the wall of ventricle was thickened (Figure 1(c)) in the TAC group. A significant difference was found in the left ventricular apical biopsy between the control group and the TAC group (Figure 1(b), HE staining 400x magnification). The single ventricular myocytes in the TAC group were larger than those in the control group (Figure 1(d)). We evaluated the cardiac systolic and diastolic functions by measuring the left ventricular posterior wall thickness (LPWD) of the control group and the TAC group (Figure 1(e)). Compared with the control group, the LPWD of the TAC group was significantly increased (0.22 ± 0.02 cm versus 0.31 ± 0.03 cm, *n* = 10, *P* < 0.01, Figure 1(e3)).

### 3.2. Effects of WXKL on the APs in the Control Group and the TAC Group

**Figure 2:**
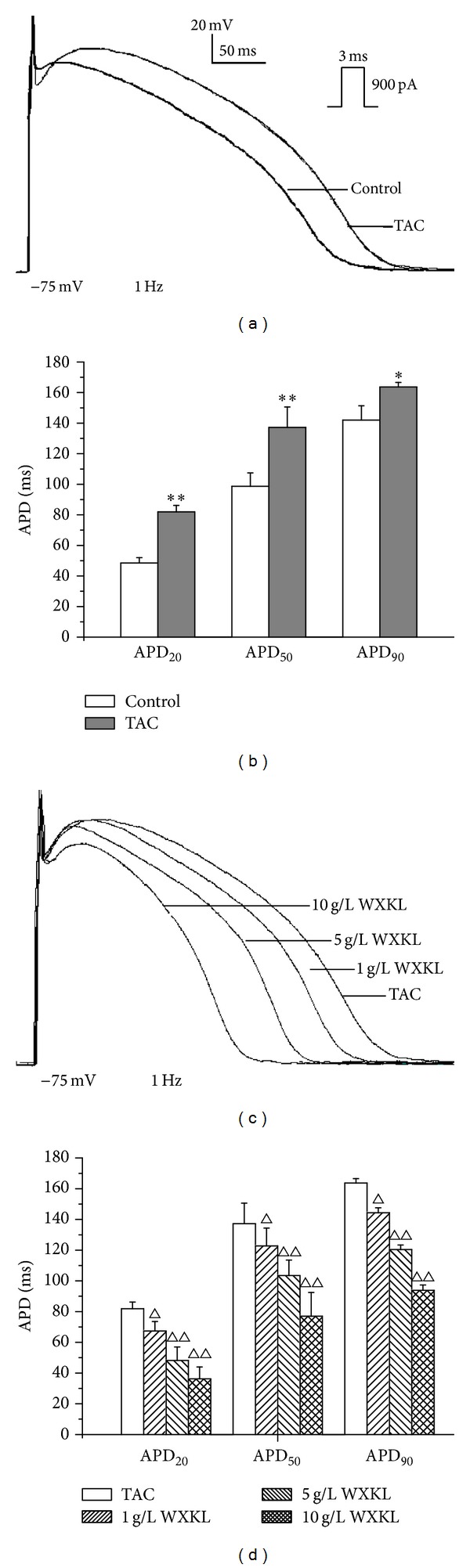
Representative AP traces recorded from the control and the TAC group and effects of different concentrations of WXKL on the APs in the TAC group. (a) APs of the control group and the TAC group. (b) APD_20_, APD_50_, and APD_90_ of the control group and the TAC group. (c) Effects of 1, 5, and 10 g/L WXKL on the APs in the TAC group. (d) APD_20_, APD_50_, and APD_90_ of the TAC group after treatment with 1, 5, and 10 g/L WXKL. *P < 0.05 and **P < 0.01 versus the control group. ^Δ^ *P* < 0.05 and ^ΔΔ^ *P* < 0.01 versus the TAC group.

The APs were recorded by applying a 900-pA current pulse with duration of 3 ms at 1 Hz in the current-clamp mode. The APD was significantly prolonged in the TAC group compared with the control group (Figure 2(a)). The APDs obtained with 20%, 50%, and 90% repolarisation (APD_20_, APD_50_, and APD_90_ in ms) in the control group and the TAC group were the following: 48.5 ± 3.5 ms versus 81.9 ± 4.3 ms (*n* = 6, *P* < 0.01), 98.7 ± 8.8 ms versus 137.2 ± 13.4 ms (*n* = 6, *P* < 0.01), and 142.0 ± 9.3 ms versus 163.7 ± 2.9 ms (*n* = 6, *P* < 0.05), respectively (Figure 2(b)).

After treatment with different dose of WXKL, the APD in the TAC group exhibited significant changes (Figure 2(c)). The APD_20_, APD_50_, and APD_90_ in the TAC group were markedly shortened by WXKL in a dose-dependent manner. After perfusion with 1 g/L WXKL, the APD_20_, APD_50_, and APD_90_ were shortened from 81.9 ± 4.3 ms to 67.4 ± 6.2 ms (*n* = 6, *P* < 0.05), 137.2 ± 13.4 ms to 122.8 ± 11.6 ms (*n* = 6, *P* < 0.05), and 163.7 ± 2.9 ms to 144.4 ± 3.2 ms (*n* = 6, *P* < 0.05), respectively. When treated with 5 g/L WXKL, the APD_20_, APD_50_, and APD_90_ were shortened from 81.9 ± 4.3 ms to 48.2 ± 8.8 ms (*n* = 6, *P* < 0.01), 137.2 ± 13.4 ms to 103.5 ± 10.1 ms (*n* = 6, *P* < 0.01), and 163.7 ± 2.9 ms to 120.4 ± 3.0 ms (*n* = 6, *P* < 0.01), respectively. When treated with 10 g/L WXKL, the APD_20_, APD_50_, and APD_90_ were shortened from 81.9 ± 4.3 ms to 36.2 ± 7.8 ms (*n* = 6, *P* < 0.01), 137.2 ± 13.4 ms to 77.0 ± 15.5 ms (*n* = 6, *P* < 0.01), and 163.7 ± 2.9 ms to 93.9 ± 3.4 ms (*n* = 6, *P* < 0.01), respectively (Figure 2(d)). To understand the mechanisms that were responsible for the observed changes in the APD in the TAC group, we examined the *I*_Ca-L_ in the control group and the TAC group.

### 3.3. Effects of WXKL on the *I*_Ca-L_ in the Control Group

**Figure 3:**
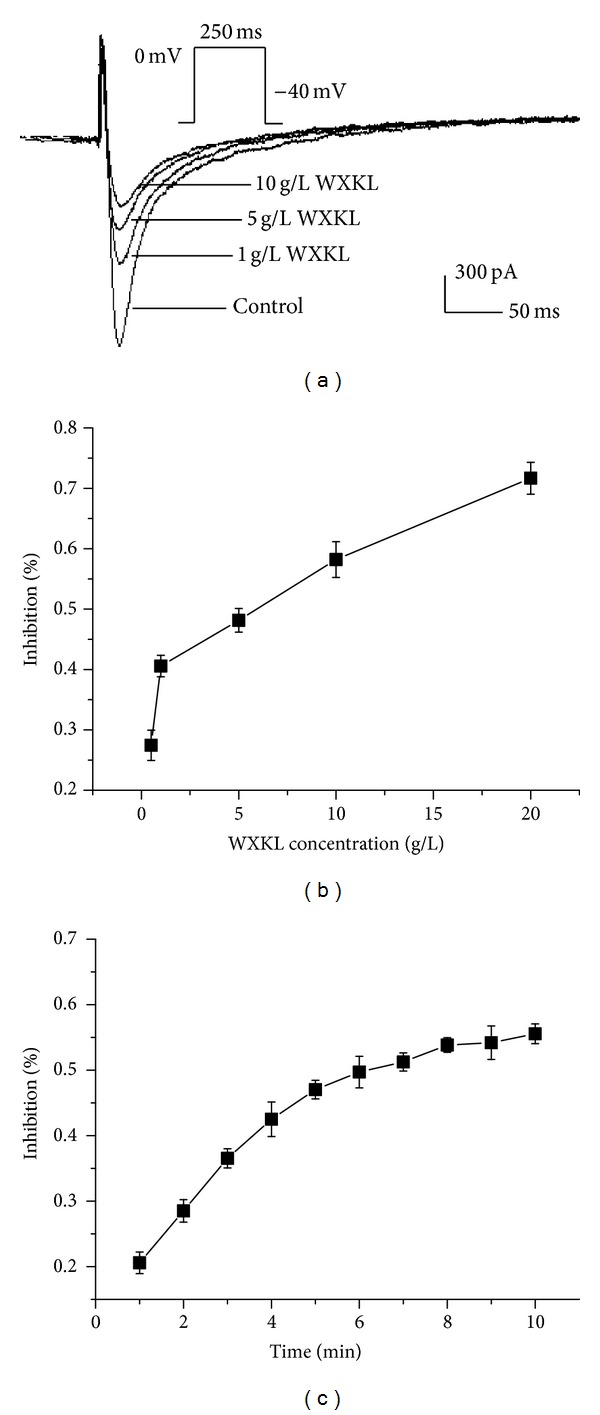
Effects of WXKL on the *I*_Ca-L_ in the control group. (a) Effects of 1, 5, and 10 g/L WXKL on the *I*_Ca-L_ in the control group. After treatment with WXKL, the current amplitudes of the control group were significantly reduced. (b) Concentration-dependent effects of WXKL on the *I*_Ca-L_ in the control group (IC_50_ = 6.23 g/L). (c) Time-dependent effects of 5 g/L WXKL on the *I*_Ca-L_ in the control group. *P < 0.05 and **P < 0.01 versus the control group.

To avoid the influence of current rundown, all of the recordings were obtained within 25 minutes. The current traces were obtained during a depolarising pulse from the holding potential of −40 mV to 0 mV over 250 ms. After WXKL (0.5, 1, 5, 10, and 20 g/L) treatment, the *I*_Ca-L_ in control group significantly decreased by 27.45 ± 2.51%, 40.57 ± 1.77%, 48.15 ± 1.95%, 58.22 ± 2.96%, and 71.68 ± 2.63%, respectively (*n* = 10, *P* < 0.01, Figure 3(a)). Thus, we concluded that WXKL significantly decreased the *I*_Ca-L_ of the control group in a concentration-dependent manner. The IC_50_ of WXKL was found to be 6.23 g/L (Figure 3(b)). The time-dependent curve showed that the effects of WXKL on the *I*_Ca-L_ remained stable after 5 minutes (Figure 3(c)).

### 3.4. Effects of WXKL on the *I*_Ca-L_ in the TAC Group

**Figure 4:**
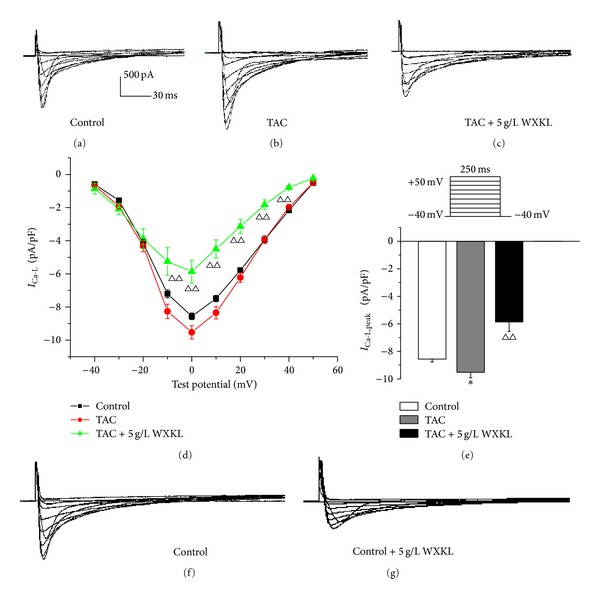
Effects of WXKL on the *I*_Ca-L_ in the TAC group. (a) Representative *I*_Ca-L_ traces recorded from the control group. (b) Representative *I*_Ca-L_ traces recorded from the TAC group. (c) Representative *I*_Ca-L_ traces recorded from the TAC group in the presence of 5 g/L WXKL. (d) The peak current density-voltage relationship showed that the current densities in the TAC group were significantly increased by a range of −10 mV to +10 mV, and the current densities in the TAC group after treatment with 5 g/L WXKL were significantly reduced by a range of −10 mV to +40 mV. (e) The peak current densities in each group exhibited significant differences. ((f) and (g)) Representative *I*_Ca-L_ traces recorded from the control group and after treatment with 5 g/L WXKL. *P < 0.05 and **P < 0.01 versus the control group. ^Δ^ *P* < 0.05 and ^ΔΔ^ *P* < 0.01 versus the TAC group.

The current-voltage (*I-V*) curves were obtained by applying voltage steps in 10-mV increments (−40 mV to +50 mV) for 250 ms from a holding potential of −40 mV. The representative *I*_Ca-L_ traces show that the amplitude of the *I*_Ca-L_ was higher in the TAC group compared with the control group (Figures 4(a) and 4(b)). After treatment with 5 g/L WXKL, the *I*_Ca-L_ was significantly reduced in the TAC group (Figure 4(c)). The *I-V* curves also show that the current densities in the TAC group were significantly increased by a range of −10 mV to +10 mV compared with the control group (*n* = 10, *P* < 0.01). The current densities of the TAC group after treatment with 5 g/L WXKL were significantly decreased by a range of −10 mV to +40 mV compared with untreated TAC group (*n* = 10, *P* < 0.01, Figure 4(d)). The mean current densities at 0 mV were −8.56 ± 0.20 pA/pF in the control group and −9.52 ± 0.40 pA/pF in the TAC group (*n* = 10, *P* < 0.05, Figure 4(e)). In contrast, the mean current density at 0 mV in the TAC group after treatment with 5 g/L WXKL was decreased to −5.86 ± 0.69 pA/pF (*n* = 10, *P* < 0.01, Figure 4(e)).

### 3.5. Effects of WXKL on the Steady-State Activation and Inactivation Kinetics of *I*_Ca-L_ in the TAC Group

The steady-state activation curves of *I*_Ca-L_ were determined using pulses from −40 mV to +50 mV at 10-mV increments for 250 ms. The steady-state inactivation curves of *I*_Ca-L_ were determined using pulses from −60 mV to +30 mV at 10-mV increments for 1,000 ms. The steady-state activation curves was described assuming that a Boltzmann function:

(1) $$\begin{array}{c} \left(\frac{G}{G_{\max}}=\frac{1}{1+\text{exp⁡}\left(\left(V_{1/2,\text{ack}}-V_{\text{rev}}\right)/k\right)}\right). \end{array}$$

The steady-state inactivation curves was described assuming that a Boltzmann function:

(2) $$\begin{array}{c} \left(\frac{I}{I_{\max}}=\frac{1}{1+\text{exp⁡}\left(\left(V_{\text{rev}}-V_{1/2,\text{inact}}\right)/k\right)}\right). \end{array}$$

**Figure 5:**
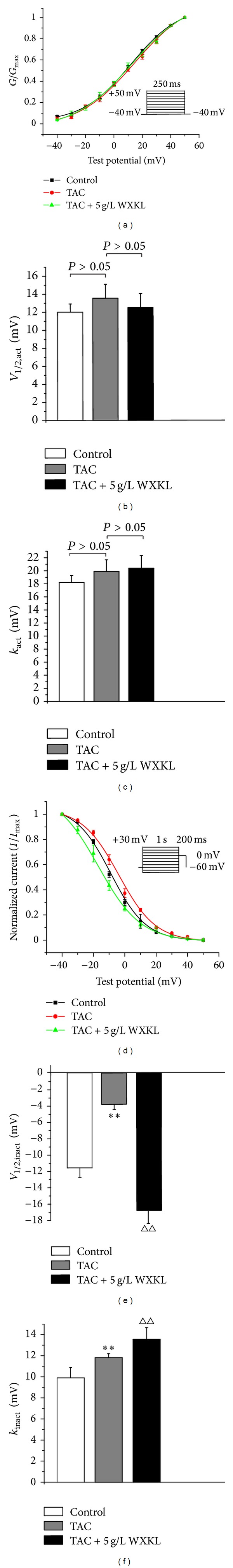
Effects of WXKL on the steady-state activation and inactivation kinetics of the *I*_Ca-L_ in the TAC group. (a) Steady-state activated curve of the control group, the TAC group and the TAC group treated with 5 g/L WXKL. The steady-state activation curve of each group did not exhibit significant differences. (b) The *V*_1/2,act_ of each group did not exhibit significant differences. (c) The *k*_act_ of each group did not exhibit significant differences. (d) The steady-state inactivation curve of the TAC group was shifted to a more negative potential, whereas the steady-state inactivation curve of the TAC group treated with 5 g/L WXKL was shifted to a more active potential. (e) The *V*_1/2,inact_ in each group exhibited significant differences. (f) The *k*_inact_ of each group exhibited significant differences. *P < 0.05 and **P < 0.01 versus the control group. ^Δ^ *P* < 0.05 and ^ΔΔ^ *P* < 0.01 versus the TAC group.

The steady-state activation curve in each group exhibited no significant difference (Figure 5(a)). The half-activation potentials (*V*_1/2,act_, at which 50% of the channels are activated) in the control and the TAC groups were 12.01 ± 0.91 mV and 13.55 ± 1.55 mV, respectively (*n* = 10, *P* > 0.05). Treatment with 5 g/L WXKL shifted the *V*_1/2,act_ from 13.55 ± 1.55 mV to 12.50 ± 1.57 mV in the TAC group (*n* = 10, *P* > 0.05, Figure 5(b)). The slope factor (*k*_act_) activation values in the control group, the TAC group, and the TAC group after treatment with 5 g/L WXKL were 18.22 ± 1.04 mV, 19.89 ± 1.79 mV, and 20.39 ± 1.96 mV, respectively. These values were not significantly different (*n* = 10, *P* > 0.05, Figure 5(c)).

However, compared with the control group, the steady-state inactivation curve in the TAC group shifted to a more positive potential. In the presence of 5 g/L WXKL, the steady-state inactivation curve of the TAC group was shifted to a more negative potential (Figure 5(d)). The half-inactivation potentials (*V*_1/2,inact_, at which 50% of the channels are inactivated) in the control and the TAC groups were shifted from −11.60 ± 1.15 mV and −3.83 ± 0.66 mV, respectively (*n* = 10, *P* < 0.01, Figure 5(e)). The half-inactivation potential in the TAC group after treatment with 5 g/L WXKL was −16.89 ± 2.24 mV (*n* = 15, *P* < 0.01, Figure 5(e)). The slope factor (*k*_inact_) inactivation values in the control group, the TAC group, and the TAC group after treatment with 5 g/L WXKL were 9.89 ± 0.98 mV, 11.81 ± 0.38 mV, and 13.55 ± 1.11 mV, respectively (*n* = 15, *P* < 0.01, Figure 5(f)). These results revealed that WXKL reduced the current by accelerating inactivation of the channels.

### 3.6. Effects of WXKL on the Recovery of *I*_Ca-L_ from Inactivation in the TAC Group

**Figure 6:**
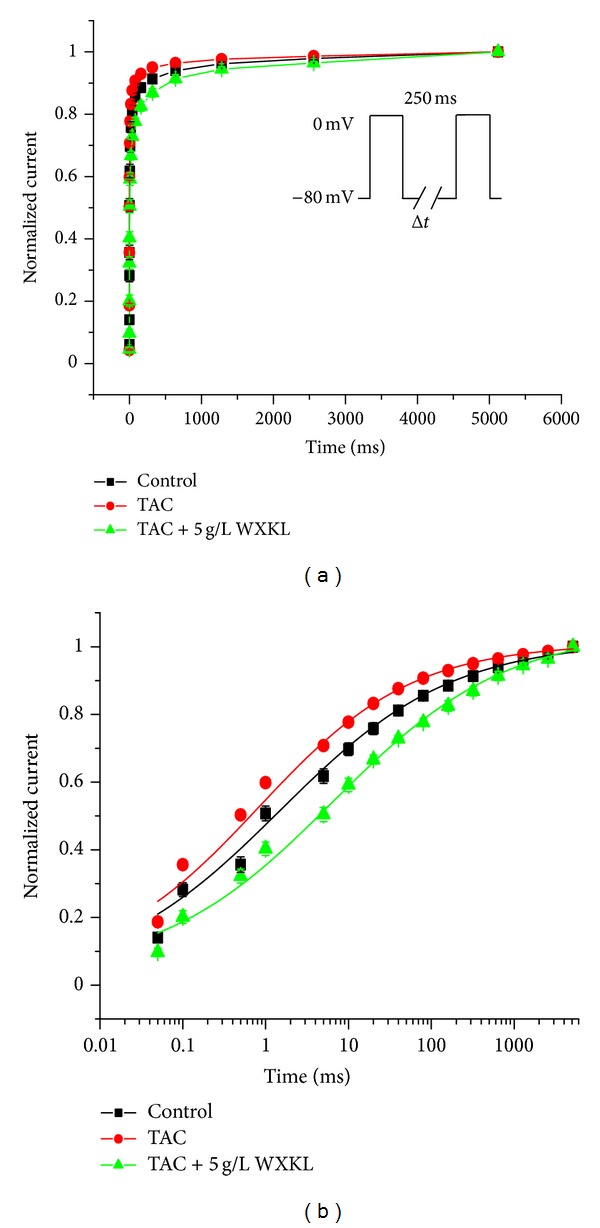
Effects of WXKL on the recovery of the *I*_Ca-L_ from inactivation in the TAC group. The time course of the recovery from inactivation was fitted with a single exponential function. The recovery from inactivation of the *I*_Ca-L_ was changed after exposure to 5 g/L WXKL.

The time course of recovery from inactivation of *I*_Ca-L_ was evaluated using a paired-pulse protocol: a conditioning pulse was first applied from a holding potential of −80 mV to 0 mV, and a test potential of 0 mV was then applied for 250 ms after various interval durations of 0.05, 0.1, 0.5, 1, 5, 10, 20, 40, 80, 160, 320, 640, 1280, 2560, and 5120 ms. The recovery curve from inactivation was fitted by a single exponential function. The treatment with 5 g/L WXKL shifted the recovery curve from the inactivation of *I*_Ca-L_ in the TAC group to the right (Figures 6(a) and 6(b)). The results showed that WXKL delayed the recovery time from inactivation.

## 4. Discussion

We can draw the following conclusions from the present study: (1) in the TAC group, WXKL treatment can significantly decrease the prolongation of the APD in a dose-dependent manner, and the APD_20_, APD_50_, and APD_90_ were all significantly shortened. (2) The amplitude of the *I*_Ca-L_ in the TAC group was increased compared with the control group, and WXKL treatment significantly reduced the *I*_Ca-L_ in the TAC group. (3) WXKL decreased the *I*_Ca-L_ by accelerating the inactivation process of the channels and delaying the recovery time from inactivation but had no significant effect on the activation process. These major findings suggest that *I*_Ca-L_ may be the target of the antiarrhythmic effect of WXKL.

Cardiac hypertrophy is a common pathological change that increases the incidence and mortality of many cardiovascular diseases. These changes are frequently induced by electrical remodelling and arrhythmogenesis. Most of the drugs that have been used can potentially induce ventricular arrhythmias. Thus, it is necessary to identify more effective and safer drugs for the treatment of arrhythmias induced by cardiac hypertrophy.

The functioning of the heart depends on the normal action potential, and the normal action potential depends on the normal functioning of ion channels. The abnormality that is most consistently found in animal models of cardiac hypertrophy is the prolongation of the APD [23–25]. Ca^2+^ plays a key role in the excitation-contraction coupling and the activation of Ca^2+^-dependent signalling pathways. The APD prolongation may increase the Ca^2+^ entry via *I*_Ca-L_ during the long plateau phase, which would cause an accumulation of Ca^2+^ in the sarcoplasmic reticulum (SR) and spontaneous SR Ca^2+^ release [26]. Increases in the intracellular Ca^2+^ concentration, which can be caused by multiple mechanisms, such as L-type Ca^2+^ channels coupled with Ca^2+^-induced Ca^2+^ release from the ryanodine receptors, and T-type Ca^2+^ channels could lead to cardiac hypertrophy [27]. However, the L-type Ca^2+^ channel is involved in the predominant mechanism responsible for the influx of Ca^2+^ in cardiac cells [28]. This channel also plays an important role in the generation of AP under physiological and pathophysiological conditions. The blockade of the L-type Ca^2+^ channels results in antiarrhythmic actions [29]. Thus, blocking the Ca^2+^ channels, reducing the Ca^2+^ overload; and weakening the myocardial contractility will benefit the treatment of cardiac hypertrophy and heart failure.

WXKL is the first antiarrhythmic Chinese medicine to be approved by the state. A large number of clinical trials have confirmed that WXKL can improve the left ventricular diastolic function and reduce the degree of left ventricular hypertrophy with high blood pressure, which ultimately leads to a reduction in the incidence of arrhythmias [30]. Most of the previous studies that analysed the antiarrhythmic properties of WXKL were based on normal myocytes or on drug-induced cardiac hypertrophy. However, it was unknown whether the drugs would exhibit the same effect on pathological myocytes, and the experimental studies on this subject were rare. During these years, an increasing number of studies have been performed using animal models of hypertrophy. A large number of studies have been performed in rats using different interventions to induce hypertrophy [31, 32]. Thus, this paper investigated the antiarrhythmic effects of WXKL on a TAC model using an electrophysiological technology.

Previous experiments have shown that WXKL can reverse cardiac hypertrophy induced by ISO. WXKL significantly reduced HW/BW, LVW/BW and the expression of *β*-catenin and e-myc. Thus, the use of WXKL for the treatment of patients with hypertension and arrhythmia may be a reasonable and effective choice [33]. Our previous study has shown that WXKL inhibits heart failure and cardiac arrhythmias via a mechanism that may involve the regulation of the CaMKII signal transduction pathway similar to amiodarone. WXKL treatment can increase the calcium transient amplitude in isolated cardiac myocytes from rats with myocardial infarction and reduce the incidence of cardiac arrhythmias in rat myocardial infarction model [34]. In our model, we found that the APD of the TAC group was significantly prolonged compared with the control group, which was in accordance with the results of previous studies [25, 35]. The APD_20_, APD_50,_ and APD_90_ were all longer than those of the control group. After WXKL treatment, the APD_20,_ APD_50,_ and APD_90_ of the TAC group were significantly shortened. WXKL abbreviated the prolongation of the APD in a dose-dependent manner. The change in the APD indicated that the ion channel currents of the TAC group were also changed. Considering the important role of Ca^2+^ in cardiac hypertrophy, we investigated the effect of WXKL on the L-type Ca^2+^ channel.

A previous study showed the effect of WXKL on the *I*_Ca-L_ and *I*_to_ in normal rat ventricular myocytes. WXKL decreased the *I*_Ca-L_, shifted the steady-state activation curve to the right, and prolonged the recovery time from inactivation [17]. However, the results from studies of changes of the *I*_Ca-L_ in cardiac hypertrophy models are inconsistent. These disparities are due in part to the differences in the models used and the variations in the experimental conditions. Our results are consistent with the studies that showed a significant increase in the Ca^2+^ current [24]. In our study, we found that both the current amplitude and the current density of the *I*_Ca-L_ in the TAC group were higher than those in the control group. The acute application of WXKL inhibited the *I*_Ca-L_ in a concentration-dependent manner in the control group. The IC_50_ was found to be 6.23 g/L. WXKL significantly decreased the peak current of the *I*_Ca-L_ in the TAC group. It appears that the effect of WXKL can significantly relieve the increase in the *I*_Ca-L_ in the TAC group. The experiments revealed that WXKL was able to block the *I*_Ca-L_, which may account for the shortening of the APD and contribute to some of its antiarrhythmic effects. WXKL significantly reduced the APD_20_, APD_50_, and APD_90_ of the TAC group. This result is well explained by the reduction of the *I*_Ca-L_. We demonstrated that WXKL has the potential to attenuate the development of cardiac hypertrophy by affecting the signalling mechanisms of cardiac myocytes. In our study, a WXKL concentration of 5 g/L was used to explore the mechanism through which this agent treats cardiac hypertrophy. This concentration is close to the IC_50_. However, the results show that the WXKL treatment decreased the *I*_Ca-L_ in the TAC group and in the control group to a level that was lower than the normal level. We should therefore use a lower dose of WXKL and analyse the resulting effects on the *I*_Ca-L_ of the TAC group. In addition, it is unclear whether an excessive dose of WXKL can exert a significant antiarrhythmic effect with fewer side effects.

Furthermore, we determined the effect of WXKL on the gating mechanism of *I*_Ca-L_ in the TAC group. The steady-state activation curves in each group were not significantly different. Compared with the control group, the steady-state inactivation curve of the TAC group was shifted to a more positive potential. In the presence of 5 g/L WXKL, the steady-state activation curve was shifted to a more negative potential. This result suggests that the voltage-dependent steady-state inactivation of the L-type Ca^2+^ channels was accelerated. Moreover, 5 g/L WXKL shifted the recovery curve from inactivation of the *I*_Ca-L_ in the TAC group to the right. These data suggested that WXKL decreased the *I*_Ca-L_ through facilitation of the steady-state inactivation and retardation of the recovery from inactivation. Interestingly, we found that the effects of WXKL on the steady-state activation and inactivation procedures of the L-type Ca^2+^ channels in the TAC group were not in accordance with its effects on normal rats [17]. An explanation for this discrepancy is that pathological cells may have undergone electrical remodelling, which would change the effect that WXKL would have on these cells. The effects of WXKL on the recovery curve from inactivation of *I*_Ca-L_ were consistent and showed that the mechanism underlying the beneficial effects of WXKL may involve the regulation of the Ca^2+^ channel and a reduction in the Ca^2+^ influx. WXKL likely affects the L-type Ca^2+^ channels, alters the cellular Ca^2+^ regulation, and improves the heart function.

WXKL includes five ingredients: *Nardostachys chinensis* Batal extract, codonopsis, notoginseng, amber, and *Rhizoma Polygonati*. A study had investigated the effects of *Nardostachys chinensis* Batal extract (NcBe) on the activation kinetics of normal rat cardiac sodium channels and transient outward potassium channels. NcBe significantly blocks the *I*_Na_ and *I*_to_ of normal rat ventricular myocytes [36]. Studies could be conducted to discover more effective drugs. Furthermore, the present study focused on the effect of WXKL in the *I*_Ca-L_ of TAC rats. To investigate the mechanism through which WXKL exerts antiarrhythmic effect, it is necessary to study the related signal transduction pathways in further study. In addition to the shortening of the APD after WXKL perfusion and the excessive reduction of the *I*_Ca-L_, other ion currents in hypertrophied myocytes may be modulated by this herbal extract. Therefore, it will be valuable to obtain further insight into the effect of WXKL on the regulation of other ion channels and the related signal transduction pathway.

In summary, the present study evaluated the electrophysiologic effects and antiarrhythmic potential of WXKL in hypertrophied ventricular myocytes. The results demonstrated that WXKL treats cardiac hypertrophy and cardiac arrhythmias via a mechanism that may involve the regulation of the L-type Ca^2+^ channels. WXKL treatment significantly shortened the prolongation of the APD and reduced the *I*_Ca-L_. Further studies should explore the deeper mechanism through which WXKL treats cardiac hypertrophy to ultimately offer new avenues for the prevention and treatment of this and other related diseases.
